# A two-dimensional Fe-doped SnS_2_ magnetic semiconductor

**DOI:** 10.1038/s41467-017-02077-z

**Published:** 2017-12-05

**Authors:** Bo Li, Tao Xing, Mianzeng Zhong, Le Huang, Na Lei, Jun Zhang, Jingbo Li, Zhongming Wei

**Affiliations:** 1 State Key Laboratory of Superlattices and Microstructures, Institute of Semiconductors, Chinese Academy of Sciences & College of Materials Science and Opto-Electronic Technology, University of Chinese Academy of Sciences, Beijing, 100083 China; 2 Department of Applied Physics, School of Physics and Electronics, Hunan University, Changsha, 410082 China; 3 Fert Beijing Institute, School of Electronic and Information Engineering, BDBC, Beihang University, Beijing, 100191 China; 4 School of Materials and Energy, Guangdong University of Technology, Guangzhou, Guangdong, 510006 China

## Abstract

Magnetic two-dimensional materials have attracted considerable attention for their significant potential application in spintronics. In this study, we present a high-quality Fe-doped SnS_2_ monolayer exfoliated using a micromechanical cleavage method. Fe atoms were doped at the Sn atom sites, and the Fe contents are ∼2.1%, 1.5%, and 1.1%. The field-effect transistors based on the Fe_0.021_Sn_0.979_S_2_ monolayer show n-type behavior and exhibit high optoelectronic performance. Magnetic measurements show that pure SnS_2_ is diamagnetic, whereas Fe_0.021_Sn_0.979_S_2_ exhibits ferromagnetic behavior with a perpendicular anisotropy at 2 K and a Curie temperature of ~31 K. Density functional theory calculations show that long-range ferromagnetic ordering in the Fe-doped SnS_2_ monolayer is energetically stable, and the estimated Curie temperature agrees well with the results of our experiment. The results suggest that Fe-doped SnS_2_ has significant potential in future nanoelectronic, magnetic, and optoelectronic applications.

## Abstract (web summary)

2D materials can be doped with magnetic atoms in order to boost their potential applications in spintronics. Here, the authors fabricate Fe-doped SnS_2_ monolayers and show that Fe_0.021_Sn_0.979_S_2_ exhibits ferromagnetic behaviour with perpendicular anisotropy at 2 K, and a Curie temperature of 31 K.

## Introduction

Two-dimensional (2D) layered transition metal dichalcogenides (TMDs), e.g., MoS_2_, WS_2_, and SnS_2_, are promising functional materials due to their peculiar structural and electronic properties^1–10^. Significant efforts, such as doping, strain, and chemical functionalization, have been used to obtain distinctive optical and electrical properties by tuning the band alignments of 2D materials^11–14^. Doping, which is the intentional introduction of impurities into a material, plays a significant role in functionalizing 2D materials by changing the intrinsic properties of pristine atomic layers^15–17^. For example, wolfram and selenium chemical doping of MoS_2_ is an effective way to engineer the optical bandgap^18–23^, and Nb-, Co-, and Mn-doped MoS_2_ few layers exhibit diverse transport properties^11, 12, 24^. Magnetic atom (e.g., Mn, Fe, Co, and Ni)-doped 2D TMDs are promising as 2D-diluted magnetic semiconductors (DMS)^25–29^, and many have been predicted to exhibit ferromagnetic behavior at room temperature^25, 27^. DMS, such as Mn-doped InAs and GaAs, have distinctive physical properties and provide the possibility of electronic control of magnetism^30–33^. To date, Co- and Mn-doped MoS_2_ nanosheets have been synthesized via the chemical vapor deposition method, and understanding their magnetic properties requires more research^11, 24^. Recently, Zhang et al. and Xu et al. reported the magnetic properties of Cr_2_Ge_2_Te_6_ and CrI_3_ monolayers via high-resolution and high-sensitivity magneto-optic microscopy^34, 35^. However, further investigation into the magnetism and functional properties of high-quality magnetic atom-doped 2D TMDs is warranted.

In this work, we synthesized different Fe-doped SnS_2_ (Fe_0.021_Sn_0.979_S_2_, Fe_0.015_Sn_0.985_S_2_, and Fe_0.011_Sn_0.989_S_2_) bulk crystals via a direct vapor-phase method, and we obtained Fe_0.021_Sn_0.979_S_2_ monolayer flakes via mechanical exfoliation. Monolayer Fe_0.021_Sn_0.979_S_2_ exhibits high-quality optoelectronic properties. Magnetic measurements show that Fe_0.021_Sn_0.979_S_2_ exhibits ferromagnetic behavior with a perpendicular anisotropy at 2 K and a Curie temperature of ∼31 K. The experimental results agree well with the theoretical calculations.

## Results

### Characterization of Fe-doped SnS_2_

**Fig. 1:**
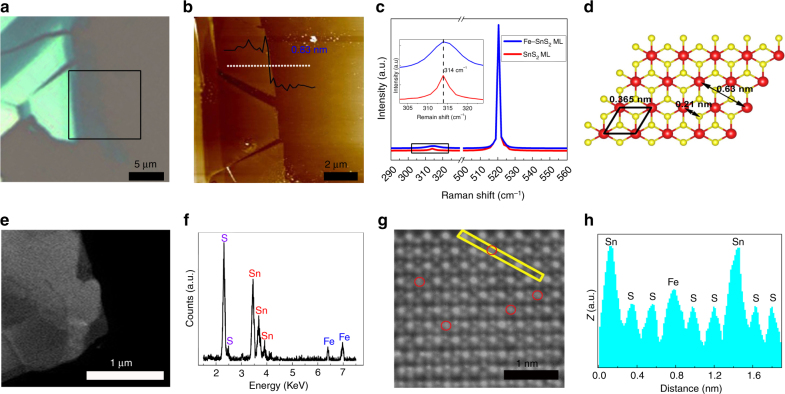
Characterization of the Fe_0.021_Sn_0.979_S_2_ flakes. **a** Optical image of the Fe_0.021_Sn_0.979_S_2_ flake. **b** AFM and **c** Raman spectra of the Fe_0.021_Sn_0.979_S_2_ and SnS_2_ monolayers. The height of the flake in **b** was obtained along the white dotted line. The inset in **c** shows the expanded view of the Raman spectra around the *A*_1*g*_ mode of the Fe_0.021_Sn_0.979_S_2_ and SnS_2_ monolayers. **d** SnS_2_ atomic structure. **e** Low-resolution HAADF-STEM image of the Fe_0.021_Sn_0.979_S_2_ flake. **f** EDS of the Fe_0.021_Sn_0.979_S_2_ flake. **g** High-resolution STEM image of the Fe_0.021_Sn_0.979_S_2_ flake; the red circles are Fe atoms. **h** *Z*-contrast mapping in the areas marked with yellow rectangles in **g**

Figure 1a shows an optical image of a mechanically exfoliated Fe_0.021_Sn_0.979_S_2_ flake on a Si/SiO_2_ substrate. Atomic force microscopy (AFM) and magnetic force microscopy (MFM) were used to characterize the height and magnetism of the samples, respectively. MFM is a valuable tool that can potentially be used to detect magnetic interactions between a magnetized AFM tip and samples^36^. Recently, MFM has been employed to characterize the magnetic response of single- or few-layer 2D nanosheets, such as graphene and MoS_2_^37, 38^. However, it has been reported that MFM signals have nonmagnetic contributions due to capacitive and electrostatic interactions between the nanosheets and the conductive cantilever tip^38, 39^. In this study, AFM and MFM images of Fe_0.021_Sn_0.979_S_2_ and pure SnS_2_ were obtained under the same test condition. The AFM images show that the obtained Fe_0.021_Sn_0.979_S_2_ and pure SnS_2_ are monolayers (Fig. 1b, Supplementary Fig. 1a). The MFM images show that the Fe_0.021_Sn_0.979_S_2_ monolayer has a larger negative phase shift (523 m^o^) than that of the pure SnS_2_ monolayer (51 m^o^) (Supplementary Fig. 1b, c) by approximately ten times, which should largely contribute to the difference in the magnetic and electrical properties between the Fe_0.021_Sn_0.979_S_2_ and SnS_2_ monolayers. Raman spectroscopy has been widely used in 2D TMD alloys, and it changes with the composition of the alloy^18, 40^. The Raman peaks of the Fe_0.021_Sn_0.979_S_2_ and pure SnS_2_ monolayers are located at 314 cm^−1^, corresponding to the *A*_1*g*_ mode of SnS_2_^3, 10^. The *A*_*1g*_ mode of the Fe_0.021_Sn_0.979_S_2_ monolayer is broader than that of the pure SnS_2_ monolayer (Fig. 1c). This broadening behavior, which is due to the doped atoms, has also been observed in other 2D alloys^20^.

The crystallinity of the Fe_0.021_Sn_0.979_S_2_ was further characterized using high-angle annular dark-field scanning transmission electron microscopy (HAADF-STEM) and transmission electron microscopy (TEM). Figure 1e shows a low-resolution HAADF-STEM image of a few layers of Fe_0.021_Sn_0.979_S_2_. Energy-dispersive X-ray spectroscopy (EDS) of the nanosheet (Fig. 1f) shows that the nanosheet contains S, Sn, and Fe elements. Using a high-resolution STEM image and the *Z*-contrast intensity distribution, the Sn (*Z* = 50), Fe (*Z* = 26), and S (*Z* = 16) atoms were directly distinguished, as shown in Fig. 1g, h. Fe atoms were doped at the Sn atom sites. The Sn–S distance is 0.22 nm (Fig. 1h), which agrees well with the theoretical value of 0.21 nm (Fig. 1d). The low-resolution TEM image (Supplementary Fig. 2a) shows a part of the few-layer Fe_0.021_Sn_0.979_S_2_ flake on the holey carbon TEM grid. The selected area electron diffraction pattern and the corresponding high-resolution TEM image reveal that this flake has a high-quality hexagonal symmetry structure, and the lattice spacing of (100) plane is 0.311 nm (Supplementary Fig. 2b, c). The elemental mapping images from EDS show that Sn, S, and Fe elements are uniformly distributed throughout the entire flake (Supplementary Fig. 2d–f).

X-ray photoelectron spectroscopy (XPS) is a powerful tool for understanding the chemical states and composition of elements that exist within a material. The binding energy values obtained in the XPS analysis of Fe_0.021_Sn_0.979_S_2_ were corrected by referencing the C 1*s* peak to 284.7 eV (Supplementary Fig. 3a). The binding energies of the Sn *3d*_5/2_ and S 2*p*_3/2_ electron peaks are 486.7 eV and 161.6 eV (Supplementary Fig. 3b, c), respectively, which is consistent with the previously reported values for SnS_2_^41^. The binding energy of the Fe 2*p*_3/2_ electron peak is 712.2 eV (Supplementary Fig. 3d), which is close to the Sn *3p* electron peak (716 eV). The binding energy of the Fe 2*p*_3/2_ electron in this instance is obviously larger than the reported values of other iron compounds (the binding energies of Fe 2*p*_3/2_ in Fe_2_O_3_, FeCl_3_, and FeCl_2_ are 710 eV, 711.5 eV, and 710.6 eV, respectively)^42^. In general, the binding energy of the same electron from one atom grows with increasing oxidation state, which is related to the bonding hybridization with its nearest-neighbor atoms. In this experiment, Fe atoms were doped by substituting Sn sites (Fig. 1g), and each Fe atom is surrounded by six S atoms to form an octahedral coordination. The oxidation state of the Fe atom should be +4, leading to a high binding energy for Fe 2*p*_3/2_ in Fe_0.021_Sn_0.979_S_2_. Furthermore, quantitative analysis of the XPS spectra reveals that the content of Fe is ∼2.1% in Fe_0.021_Sn_0.979_S_2_.

### Electronic and optoelectronic properties of Fe-doped SnS_2_

**Fig. 2:**
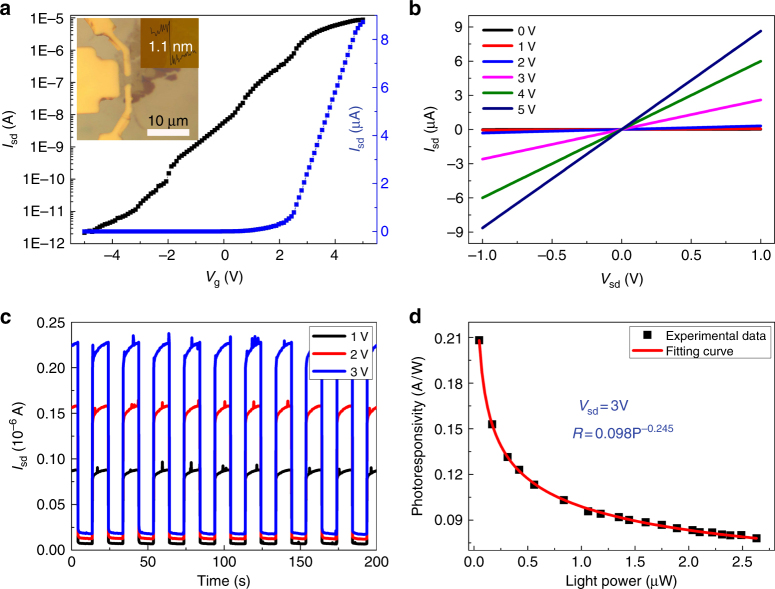
Electrical characteristics and photoresponse of the Fe_0.021_Sn_0.979_S_2_ monolayer. **a** Transfer and **b** output characteristics of Fe_0.021_Sn_0.979_S_2_. The inset shows an optical image of one typical device and the AFM image of the corresponding sample used for fabricating the device. **c** Time-dependent *I*_sd_ of the transistor based on Fe_0.021_Sn_0.979_S_2_ during the light (638 nm, 2.63 μW) switching on/off under a positive source–drain voltage, *V*_sd_, from 1 to 3 V. **d** Photoresponsivity (*R*) as a function of light power (*P*) with a *V*_sd_ of 3 V

To study the electronic transport property and photoresponse of the Fe_0.021_Sn_0.979_S_2_ monolayer on the Al_2_O_3_/Si substrate, we fabricated field-effect transistors (FETs) from exfoliated Fe_0.021_Sn_0.979_S_2_ (Fig. 2a). The device characteristics of the FETs were measured at room temperature. Figure 2a, b shows the typical transfer and output characteristics of Fe_0.021_Sn_0.979_S_2_, respectively, achieving an excellent n-type behavior. As the gate voltage (*V*_g_) varied from −5 to 5 V, the source–drain current (*I*_sd_) changed from 1.1 × 10^−12^ A to 8 × 10^−6^ A, corresponding to a high on/off current ratio of 7.3 × 10^6^. The field-effect mobility can be obtained using the formula

1 $$\mu = \frac{{\partial I_{{\mathrm{sd}}}}}{{\partial V_{\mathrm{g}}}}\left( {\frac{L}{{WC({\mathrm{Al}}_{\mathrm{2}}{\mathrm{O}}_3)V_{{\mathrm{sd}}}}}} \right),$$

where *L* and *W* are the length and width of the device, and *C*(Al_2_O_3_) is the Al_2_O_3_ gate capacitance, which can be given by equation *C*(Al_2_O_3_)=*ε*_0_ *ε*_r_/*d*. Thus, *ε*_0_ (8.85 × 10^−12^ Fm^−1^) is the vacuum dielectric constant, and *ε*_r_ (6.4) and *d* (30 nm) are the dielectric constant and thickness of Al_2_O_3_, respectively. Based on the transport curve (Fig. 2a), the calculated electron mobility is 8.15 cm^2^ V^−1^ s^−1^. Fe_0.021_Sn_0.979_S_2_ and Fe_0.021_Sn_0.979_S_2_ were also synthesized by modulating the growth conditions (Supplementary Fig. 4). The electronic transport properties of Fe_0.021_Sn_0.979_S_2_ (Supplementary Fig. 5) were investigated, and the calculated mobilities are larger than the values for pure SnS_2_ (Supplementary Table 1). The mobility increases with the Fe content in the samples. The output curves show that the *I*_sd_ is linear at low *V*_sd_, and the sample had a good contact with the electrode (Fig. 2b). The mobility is related to the mean-free time (*τ*) and effective mass (*m* ^*^) of the electron as follows:

2 $$\mu = \frac{{q\tau }}{{m^{\mathrm{*}}}}.$$

In 2D-doped TMDs, the mean-free time of the electron is decreased for enhanced carrier scattering, and the mobility decreases. Thus, searching for a decreased electron effective mass for 2D-doped TMDs is a feasible route to improve the mobility. The density functional theory (DFT)-calculated *m* ^*^ values at the conduction band bottom of monolayer SnS_2_ and Fe–SnS_2_ are listed in Supplementary Table 2. The *m* ^*^of Fe–SnS_2_ is smaller than that of pure SnS_2_, which partly contributes to the higher field-effect mobility (Supplementary Fig. 6). The stability of 2D atomic layer materials is critical for their future application. The electrical property of a typical Fe_0.021_Sn_0.979_S_2_ monolayer FET on a SiO_2_/Si substrate stored in air was measured for 1 month. After 1 month, the mobility changed from 6.1 cm^2^ V^−1^ s^−1^ to 4.7 cm^2^ V^−1^ s^−1^, and the on/off ratio changed from 1.2 × 10^6^ to 7 × 10^5^ (Supplementary Fig. 7). The results show that Fe_0.021_Sn_0.979_S_2_ is very stable and has a significant potential application in optoelectronics.

Few-layer SnS_2_ has been demonstrated as ultrasensitive photodetectors based on previous reports^3^. In this study, the photoresponsive properties of the Fe_0.021_Sn_0.979_S_2_ monolayer were examined using a 638-nm laser at room temperature. Figure 2c shows a photo on/off ratio of ∼10, and the *I*_sd_ can quickly and repetitively change between on and off states. The photoresponsivity, *R*, was obtained by using the formula *R* = *I*_ph_/*P*. *I*_ph_ is the photocurrent defined as *I*_ph_ = *I*_light_–*I*_dark_, and *P* is the light power. The photoresponsivity (*R*) shows a strong dependence on light power (*P*), and the experimental data are fitted by the equation *R* = *aP* ^*α*−*1*^. In our experiment, the fitted parameters were *a* = 0.098 and *α* = 0.755 (Fig. 2d). The maximum *R* is 206 mA W^−1^ (*P* = 57 nW), which is larger than the reported average values of few-layer SnS_2_ (Supplementary Table 1). The photocurrent response time of the Fe_0.021_Sn_0.979_S_2_ is ∼9 ms, and the response time of pure SnS_2_ is ∼6 ms (Supplementary Fig. 8). Under illumination, the impurity levels will lightly promote electron–hole recombination and increase the response time. A detailed discussion of the optoelectronic properties of Fe–SnS_2_ is provided in Supporting Information (Supplementary Fig. 9, Supplementary Note 1).

### Magnetic properties of the Fe-doped SnS_2_

**Fig. 3:**
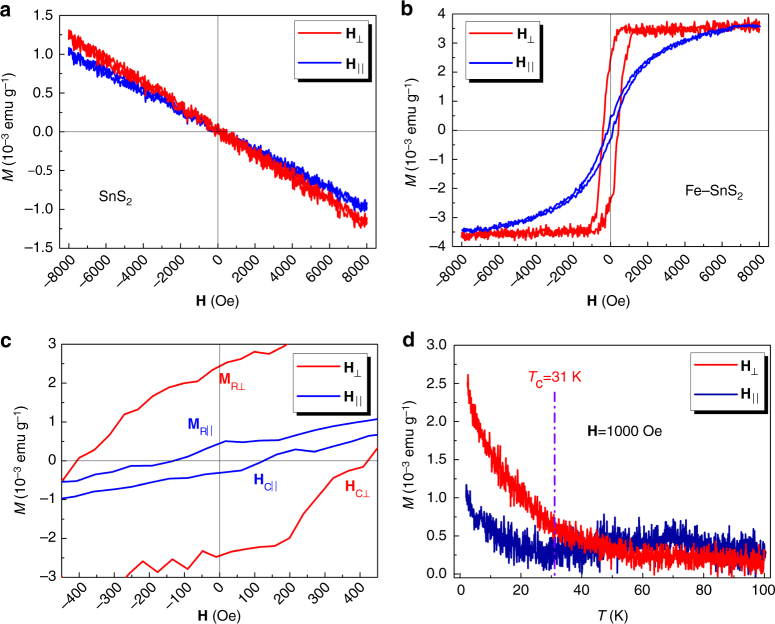
Magnetization data for SnS_2_ and Fe_0.021_Sn_0.979_S_2_. **a**, **b** Magnetic hysteresis loops for SnS_2_ and Fe_0.021_Sn_0.979_S_2_ at 2 K using VSM, respectively. **c** Expanded view of the loop of Fe_0.021_Sn_0.979_S_2_ in **b**. **d** Magnetization as a function of temperature for Fe_0.021_Sn_0.979_S_2_ from 2 K to 100 K. The applied magnetic field was 1000 Oe

The magnetic behaviors of the SnS_2_ and Fe_0.021_Sn_0.979_S_2_ single-crystal sheets were investigated using a vibrating sample magnetometer (VSM) from a physical properties measurement system (PPMS). The measurements were performed in two types of applied magnetic fields (**H**): perpendicular to the sheet, e.g., parallel to the [001] direction (**H**_⊥_), and parallel to the sheet, e.g., perpendicular to the [001] direction (**H**_‖_). Magnetic hysteresis loops at 2 K for SnS_2_ and Fe_0.021_Sn_0.979_S_2_ are shown in Fig. 3a, b. The pure SnS_2_ is diamagnetic at 2 K both in the **H**_⊥_ and **H**_‖_ directions because of the saturated electronic structure. The M–H curve of the Fe_0.021_Sn_0.979_S_2_ sheet shows remarkable anisotropy in the **H**_‖_ and **H**_⊥_ directions at 2 K, respectively. The saturation magnetization (**M**_S_), coercivity (**H**_C_), and remnant magnetization (**M**_R_) values in the **H**_⊥_ and **H**_‖_ direction for Fe_0.021_Sn_0.979_S_2_ are listed in Supplementary Table 3. The coercivity and remnant magnetization of **H**_⊥_ are approximately three and five times those of **H**_‖_, confirming that the easy axis is the [001]. The magnetism versus temperature curves show that the Curie temperature (*T*_C_) is ∼31 K (Fig. 3d). Magnetic hysteresis loops of the Fe_0.021_Sn_0.979_S_2_ bulk crystal were acquired under different temperatures (Supplementary Fig. 10), and the result shows that the Curie temperature is between 35 K and 30 K, which is consistent with the magnetism versus temperature curves (Fig. 3d). Fe cluster can be formed in the crystal when the content of Fe source increases during growth (Supplementary Fig. 11 and Supplementary Note 2). The resistivity of the Fe_0.021_Sn_0.979_S_2_ thin film as a function of different thicknesses was measured from 200 to 8 K (Supplementary Fig. 12). The temperature derivative of the measured resistivity has a transition at ∼32 K for the monolayer and 41 K for 13- and 35-nm-thick samples, which are similar to the results observed at the Curie temperature of 31 K. This resistive transition could come from the magnetic transition. Fe_0.021_Sn_0.979_S_2_ and Fe_0.015_Sn_0.985_S_2_ are ferromagnetic at 2 K, whereas Fe_0.011_Sn_0.989_S_2_ is paramagnetic at 2 K (Supplementary Fig. 13). The results demonstrate that the magnetic property of Fe–SnS_2_ varies with the Fe concentration. We discuss the detailed origin of the magnetism and the magnetic anisotropy of the Fe_0.021_Sn_0.979_S_2_ sheet below.

## Discussion

**Fig. 4:**
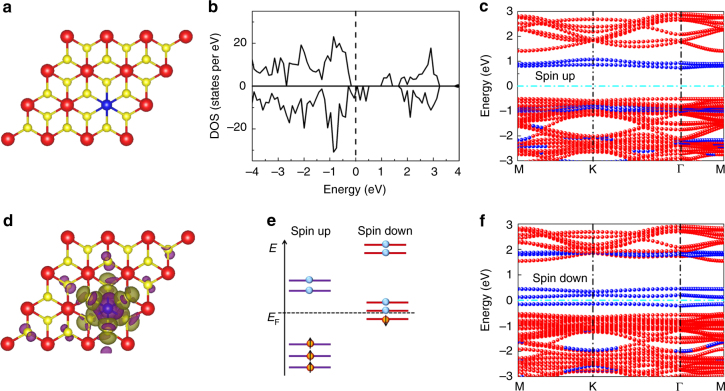
Theoretical calculations of the Fe–SnS_2_ monolayer. **a** Atomic structure of the Fe–SnS_2_ monolayer. Red, yellow, and blue balls represent Sn, S, and Fe atoms, respectively. **b** Total density of states of the Fe–SnS_2_ monolayer. **c**, **f** Projected band structures of the Fe–SnS_2_ monolayer for up-spin and down-spin channels, respectively. Red and blue circles denote the contribution of Sn and Fe atoms in the total band structure, respectively. The Fermi level was set to zero. **d** The distribution of the spin density in real space for the Fe–SnS_2_ monolayer is shown. The isosurface value was taken at 0.001 eÅ^−3^. **e** Schematic of the Fe 3*d* electron arrangement with spin in Fe–SnS_2_

Wu et al. investigated the magnetism of bulk Fe-doped SnS_2_ in detail and observed that the ferrimagnetism originates from the exchange interaction between the doped Fe atoms at the intralayer sites^27^. In this study, first-principles calculations were used to further investigate the electronic and magnetic properties of the Fe–SnS_2_ monolayer (Fig. 4a). According to the generalized gradient approximation (GGA) calculations, the bandgaps of SnS_2_ and FeS_2_ are 1.61 eV and 0.56 eV, respectively. The bandgaps of SnS_2_ and FeS_2_ are 2.07 eV and 0.97 eV, which are larger than those determined by the calculations. We tested a group of simulations with different *U* values and found that the optimized bandgap of SnS_2_ is 2.06 eV (*U* = 8.0) and the bandgap of FeS_2_ is 0.95 eV (*U* = 1.8), which are consistent with the experimental values^43, 44^. The calculated total density of states demonstrate that the Fe–SnS_2_ monolayer exhibits half-metallic behavior with 100% spin-polarized carriers at the Fermi level for the down-spin channel, whereas for the up-spin channel, the monolayer exhibits a semiconducting behavior (Fig. 4b). The projected band structures of the Fe–SnS_2_ monolayer also confirm this behavior and clearly show that the impurity levels at the Fermi level come from the Fe atom and not from the Sn atoms (Fig. 4c, f). The total magnetic moment of the Fe atom is 1.9 μ_B_, and the integration over all the occupied S 3*p* states of the S atoms bonded to the Fe dopant atom yields a magnetic moment of −0.023 μ_B_. There are six S atoms around the Fe atom, leading to a total moment of −0.14 μ_B_. The distribution of spin is shown in the spin density isosurface plot in Fig. 4d. The hybridization between the localized Fe 3*d* and the delocalized S 3*p* states leads to an antiferromagnetic (AFM) coupling between the Fe spin and S spins. When the S spins encounter one Fe, the antiferromagnetic coupling between S and Fe leads to an effective ferromagnetic (FM) structure for all of the Fe spins. Allowing for the delocalized feature of the S 3*p* states, the FM structure between the Fe spins is expected to emerge in a long range. The distribution of the outer electron of the Fe atom is 3*d* ^6^4*s* ^2^ based on crystal field theory, and we can obtain the distribution of 3*d* electrons with the spin of Fe atoms in Fe–SnS_2_ (Fig. 4e). There are three 3*d* electrons with spin-up for the Fe atom in Fe–SnS_2_, and one 3*d* electron with spin-down. The magnetic moment is 2 μ_B_, which is consistent with the calculated value of 1.9 μ_B_.

The magnetic anisotropy of Fe–SnS_2_ is due to the competition between the perpendicular and parallel spin–orbit coupling effect. The magnetic anisotropic energy (MAE) has been used to evaluate the extent of the magnetic anisotropy^45^. In this study, we assumed that the *x* and *y* direction are isotropic, and we took the *z*- and *x*-axes directions into account to analyze the anisotropy of the perpendicular and parallel direction, i.e., MAE = *E*(*x*)–*E*(*z*) with *E*(*x*) and *E*(*z*) denoting the total energy of the self-consistent calculations in the *x* and *z* magnetization directions, respectively^46, 47^. Our calculated value of MAE is 2.3 meV, confirming that the *z*-axis is the easy axis^48^. The theoretical result is consistent with the experimental result.

To further investigate the long-range FM ordering in Fe–SnS_2_, a 108-atom supercell was constructed with two Fe atoms (Supplementary Fig. 14a). The magnetic energy, Δ*E* (Δ*E* = *E*_FM_–*E*_AFM_), is −6.7 meV, showing an energetically more favorable FM coupling than an AFM coupling between the Fe spins. Supplementary Fig. 14b shows the long-range FM-ordering behavior of the Fe–SnS_2_. This behavior has also been extensively studied via theory^25–27^. The Curie temperature, *T*_C_, of the Fe–SnS_2_ monolayer can be estimated by the relation^49^:

3 $$T_{\mathrm{C}} = T_{\mathrm{b}}/{\mathrm{ln}}\left( {\frac{{3\pi T_{\mathrm{b}}}}{{4K_{\mathrm{a}}}}} \right),$$

where *T*_b_ is the bulk Curie temperature, and *K*_a_ is the anisotropy constant. The bulk magnetic energy, Δ*E*, was calculated to be −14.3 meV (Supplementary Fig. 15), and the *T*_b_ was estimated to be 56 K based on the mean-field theory and Heisenberg model^50^. *K*_a_=*K*_mca_ + *K*_sa_, where *K*_mca_ is the magnetocrystalline anisotropy constant (2.3 meV), and *K*_sa_ is the shape anisotropy constant (∼−0.17 meV^49^). Thus, *K*_a_ is 2.13 meV. The calculated *T*_C_ is ~33 K, which is consistent with the experimental result of 31 K.

An average magnetic moment for a Fe atom is ~0.020 μ_B_ per atom for Fe_0.021_Sn_0.979_S_2_ (Fig. 3b) and 0.021 μ_B_ per atom for Fe_0.015_Sn_0.985_S_2_ (Supplementary Fig. 13) at 2 K. The magnetic moment of the Fe atom is relatively low^51^, and this phenomenon should result from the AFM coupling between doped Fe atoms at the intralayer and interlayer sites. It has been reported that in magnetic atom-doped 2D TMDs, the difference in energies between FM and AFM coupling is related to the distances between magnetic atoms. When the distance between two magnetic atoms is long enough, it will be AFM coupling^25, 26, 29^. Wu et al. demonstrated that in Fe–SnS_2_, Fe atoms can have AFM coupling across the layers. In the Fe–SnS_2_ crystal^27^, Fe atoms are distributed randomly (Fig. 1g), and an AFM coupling should exist and decrease the average magnetic moment of the Fe atoms.

Field-effect transistors based on this new 2D magnetic semiconductor have a high ON/OFF ratio (>10^6^), a high electron mobility of 8.15 cm^2^ V^−1^ s^−1^, and a high photoresponsivity of 206 mA W^−1^. Pure SnS_2_ is diamagnetic, and Fe–SnS_2_ shows remarkable magnetic perpendicular anisotropic behavior with a Curie temperature of 31 K. The DFT calculations confirmed the ferromagnetic behavior and the perpendicular anisotropy of Fe–SnS_2_. The experimental and theoretical results suggest that Fe–SnS_2_ will have excellent performance for optoelectronic devices. Magnetic atom-doped 2D materials have significant potential applications in future nanoelectronic, magnetic, and optoelectronic fields.

## Methods

### Synthesis of the Fe-doped SnS_2_ bulk crystal

Fe_0.021_Sn_0.979_S_2_, Fe_0.015_Sn_0.985_S_2_, and Fe_0.011_Sn_0.989_S_2_ bulk crystals were synthesized via the direct vapor transport technique. Sn, S, and FeCl_3_ powders were mixed in stoichiometric proportions (10:30:1, 10:30:0.8, and 10:30:0.6, respectively) and placed in an ampoule. The ampoules were pumped down to the lowest-attainable pressures in our system (10^−5^ Torr) and sealed immediately to avoid blasting due to the high vapor pressure that developed inside the ampoule at growth temperatures. The ampoules were inserted into a two-zone tube furnace system, and the system was heated to 610 °C at a 30 °C h^−1^ rate. Samples were left at a constant temperature for 1 day. During the growth phase, the temperature of the growth zone was gradually lowered to 600 °C at a rate of 2 °C h^−1^, and the source temperature was maintained at 610 °C. After 5 days, the system was cooled down to room temperature at a rate of 10 °C h^−1^.

### Characterization of the Fe-doped SnS_2_

Raman measurements were performed using a Renishaw micro-Raman/PL system. STEM and TEM were performed using a JEOL2100F. AFM and MFM were performed using a Bruker AFM. Magnetic measurements were performed using a Quantum design PPMS.

### DFT calculations

According to the GGA calculations, the bandgaps of SnS_2_ and FeS_2_ are 1.61 eV and 0.56 eV, respectively. The bandgaps of SnS_2_ and FeS_2_ are 2.07 eV and 0.97 eV, which are larger than those determined via the calculations. We tested different *U* values and found that the bandgap of SnS_2_ is 2.06 eV (*U* = 8.0 on the *d* orbital) and the bandgap of FeS_2_ is 0.95 eV (*U* = 1.8 on the *d* orbital), which are consistent with the experimental values. First-principles spin-polarized calculations were performed on the basis of DFT using project or augmented wave (PAW) potentials^52^. The exchange-correlation interactions were treated by GGA with the Perdew–Burke–Ernzerhof functional^53^. The plane-wave cutoff energy was 400 eV. Monkhorst–Pack meshes of 5 × 5 × 1 and 21 × 21 × 1 were employed for geometry optimization and the calculation of density of states, respectively. To obtain reliable values for the MAEs, dense *k* points of 21 × 21 × 1 were used for the calculations.

### Data availability

The data that support the findings of this study are available from the corresponding author upon reasonable request.

## Electronic supplementary material

Supplementary Information

Peer Review File
