# Supplementary material for: A two-dimensional Fe-doped SnS2 magnetic semiconductor
Source: Nat Commun. 2017 Dec 5;8:1958. doi: 10.1038/s41467-017-02077-z (PMC5717146; doi:10.1038/s41467-017-02077-z)
Supplement: Supplementary file 1 — Supplementary Information [file 41467_2017_2077_MOESM1_ESM.pdf]

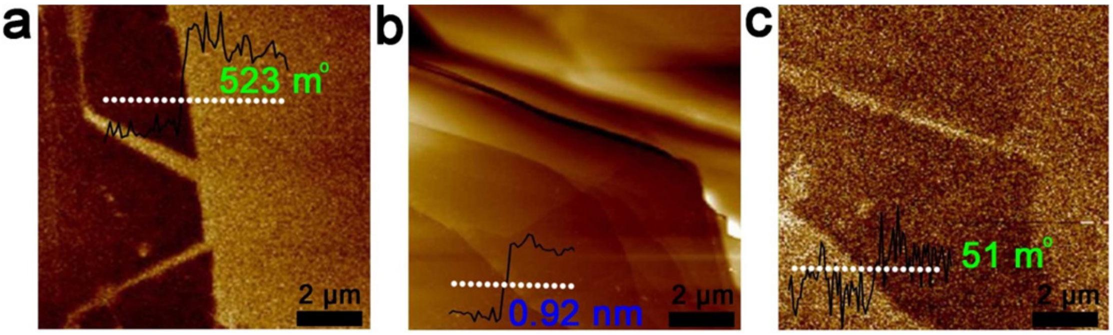

Supplementary Figure 1. (a) MFM image of $Fe_{0.021}Sn_{0.979}S_2$ flake. (b) AFM and (c) MFM images of pure SnS_2 flake. The height and phase shift are obtained along the white dotted line.

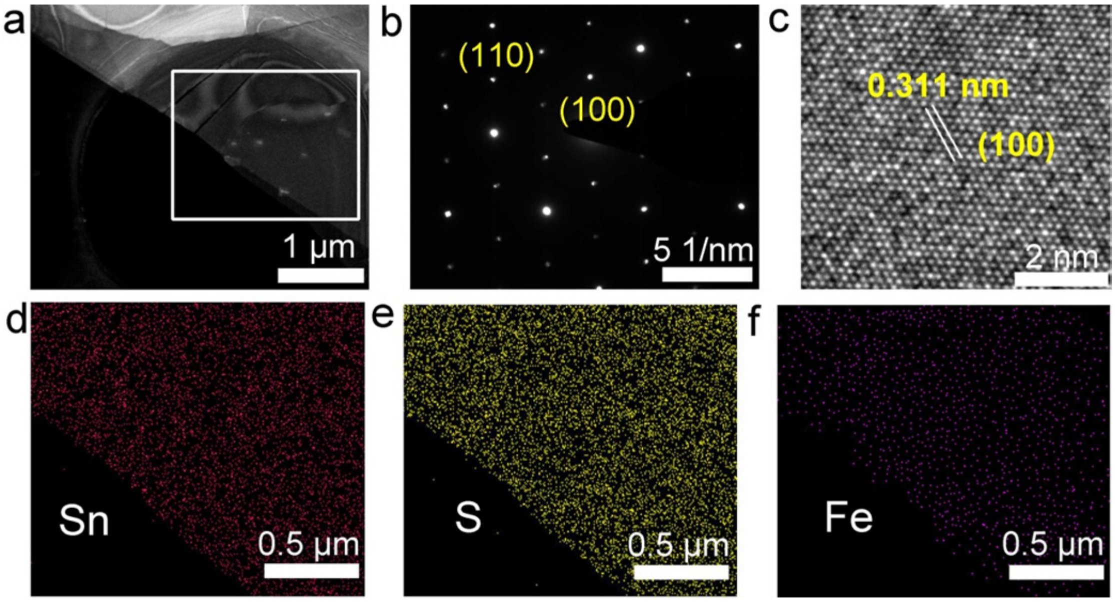

Supplementary Figure 2. TEM images and EDS elemental mapping images of $Fe_{0.021}Sn_{0.979}S_2$ flake. (a) Low-magnification TEM image, (b) SAED pattern and (c) High-magnification TEM image of $Fe_{0.021}Sn_{0.979}S_2$ flake. (d)-(f) EDS elemental mapping images of Sn, S, Fe from the marked area in (a), respectively.

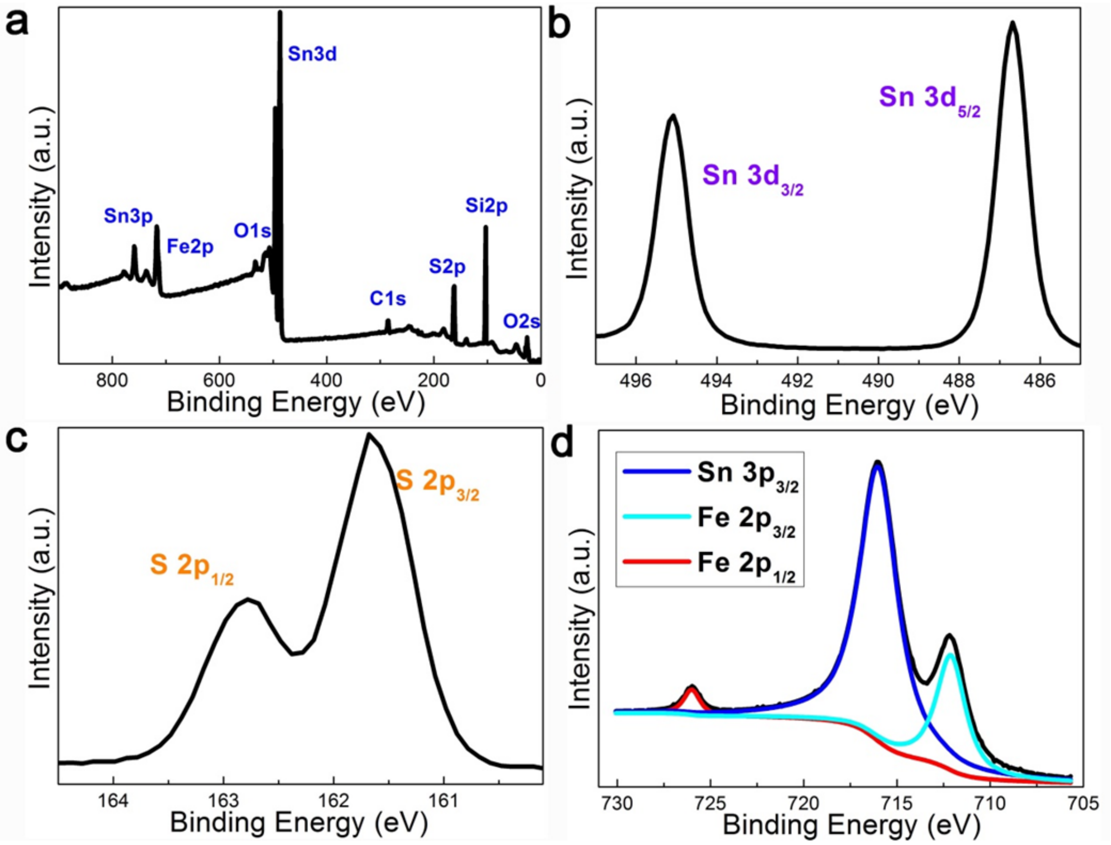

Supplementary Figure 3. XPS spectra of the exfoliated $Fe_{0.021}Sn_{0.979}S_2$ flakes on the substrate. XPS spectra of full region (a), Sn *3d* peaks (b), S *2p* peaks (c) and Sn *3p*, Fe *2p* peaks (d).

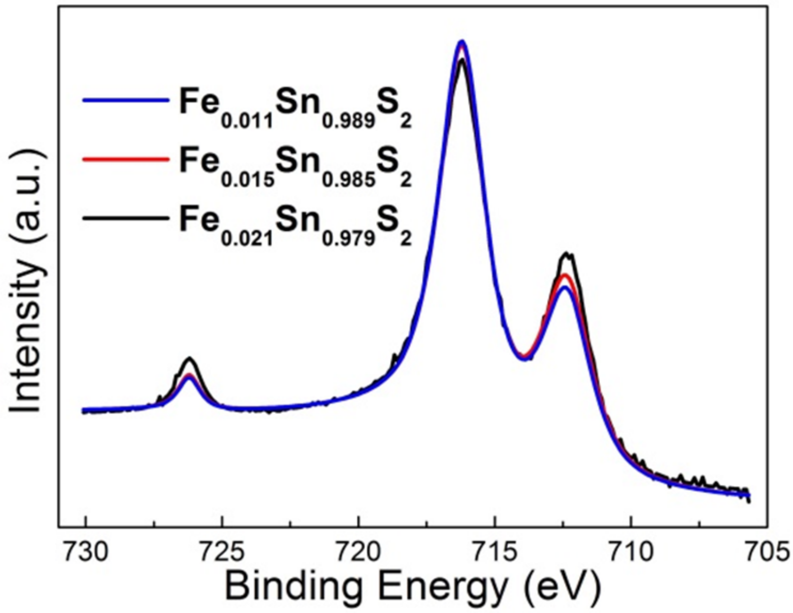

Supplementary Figure 4. XPS of different Fe doped SnS_2.

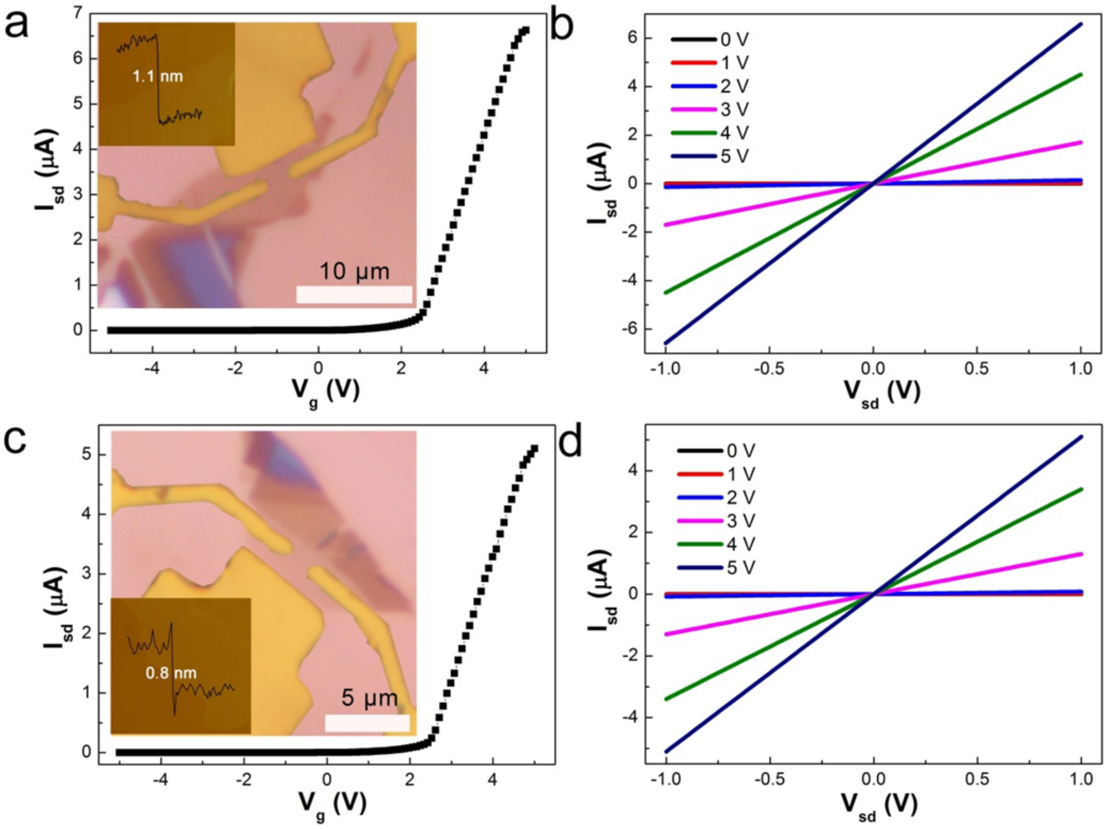

Supplementary Figure 5. Electrical characteristics and photoresponse of $Fe_{0.015}Sn_{0.985}S_2$ and $Fe_{0.011}Sn_{0.989}S_2$ monolayers. (a) Transfer and (b) Output characteristics of $Fe_{0.015}Sn_{0.985}S_2$. The inset shows the optical image of one typical device and the AFM image of the corresponding sample used for fabricating the device. (c) Transfer and (d) Output characteristics of $Fe_{0.011}Sn_{0.989}S_2$. The inset shows the optical image of one typical device and the AFM image of the corresponding sample used for fabricating the device.

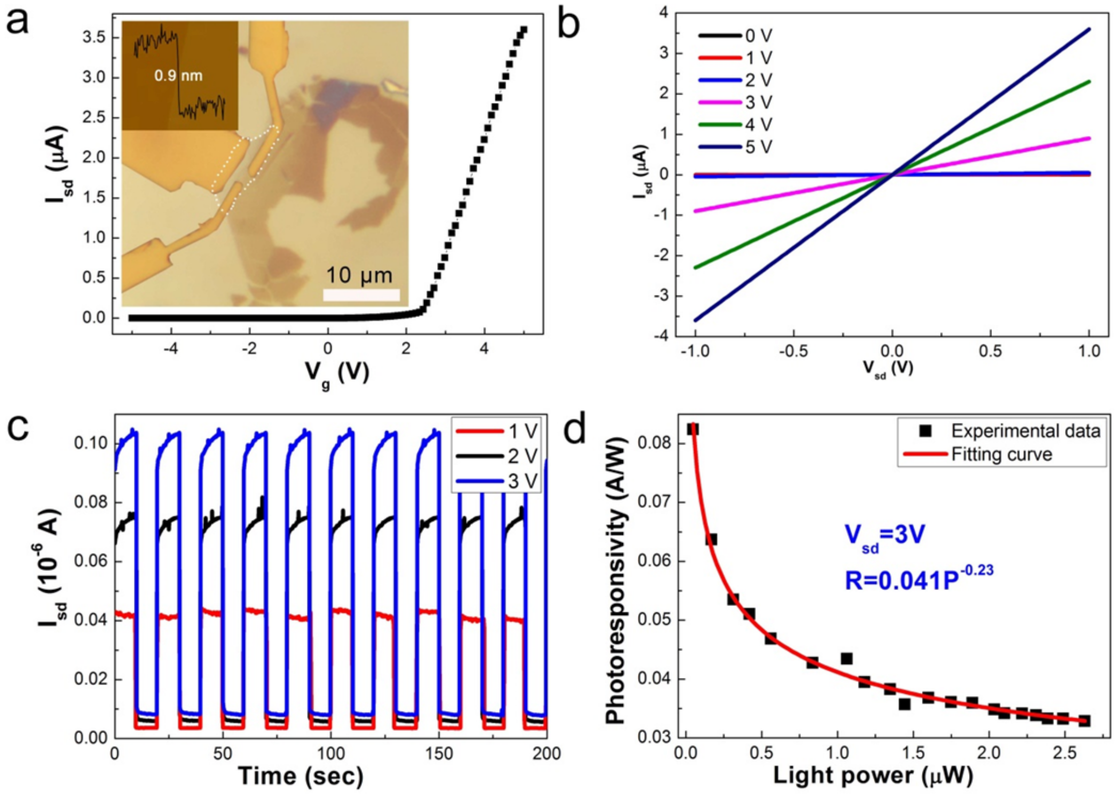

Supplementary Figure 6. Electrical characteristics and photoresponse of SnS_2 monolayer. (a) Transfer and (b) Output characteristics of SnS_2. The inset shows the optical image of one typical device and the AFM image of the corresponding sample used for fabricating the device. (c) Time dependent I_{sd} of the transistor based on the SnS_2 during the light (638 nm, 2.63 μW) switching on/off under the positive source-drain voltage V_{sd} from 1 to 3 V. (d) Photoresponsivity (R) as function of light power (P) with V_{sd} of 3V.

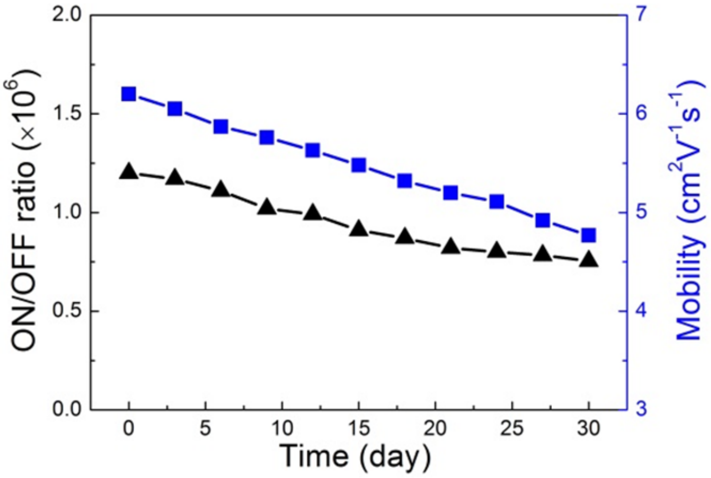

Supplementary Figure 7. ON/OFF ratio and mobility of a typical $Fe_{0.021}Sn_{0.979}S_2$ monolayer FET measured for one month.

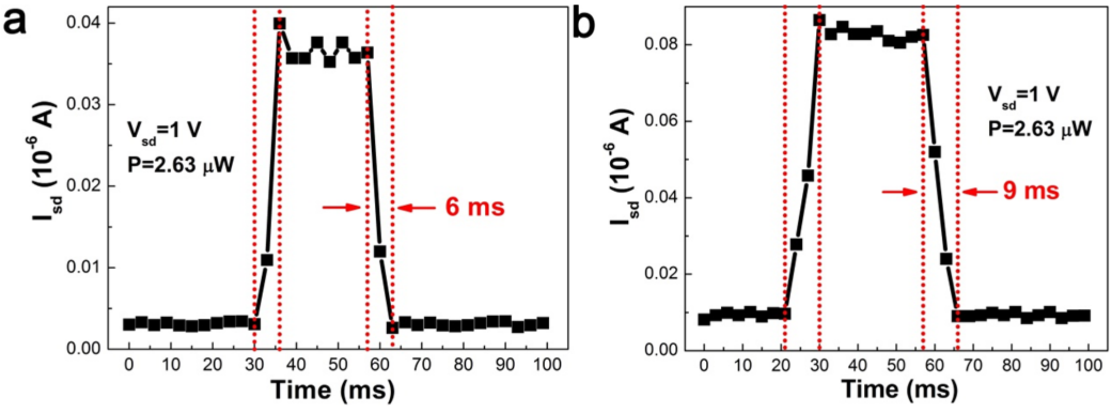

Supplementary Figure 8. High-resolution time response of (a) SnS_2 and (b) $Fe_{0.021}Sn_{0.979}S_2$ measured with V_{sd} of 1 V and light power of 2.63 μW.

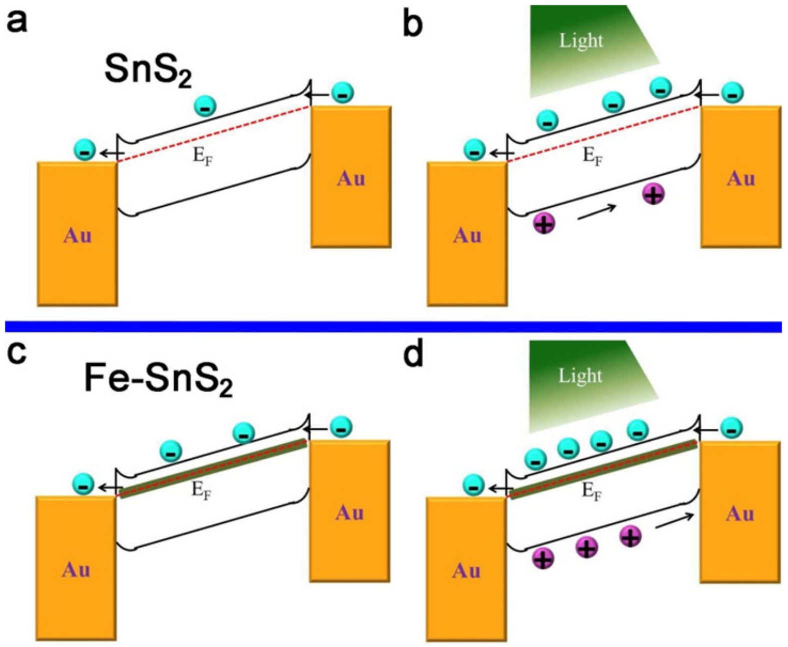

Supplementary Figure 9. Schematic of the photoconductive effect for SnS_2 and Fe-SnS_2. (a) Band alignment for SnS_2 contacted with two metals (Au) under an external bias without illumination. (b) Band alignment under illumination for SnS_2. (c) Band alignment for Fe-SnS_2 contacted with two metals (Au) under an external bias without illumination. (d) Band alignment under illumination for Fe-SnS_2.

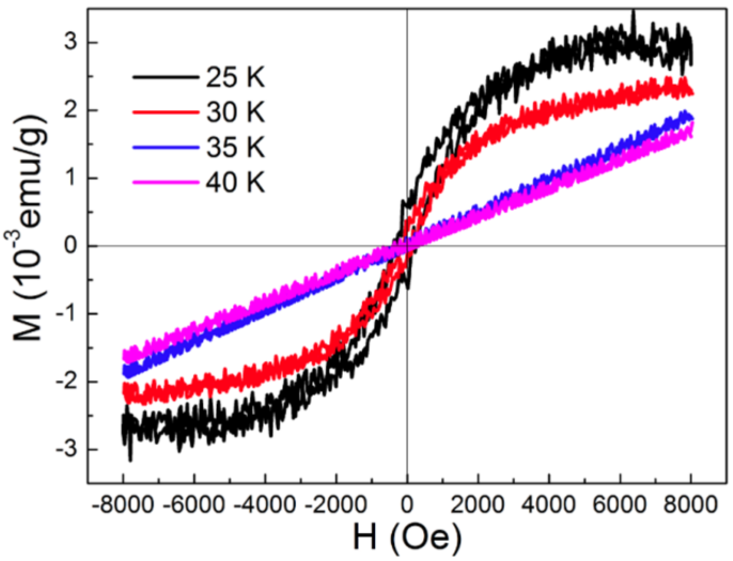

Supplementary Figure 10. Magnetic hysteresis loops for $Fe_{0.021}Sn_{0.979}S_2$ bulk at 40 K, 35 K, 30 K and 25 K using VSM, respectively. The applied magnetic field is perpendicular to the sheet ($H_{\perp}$).

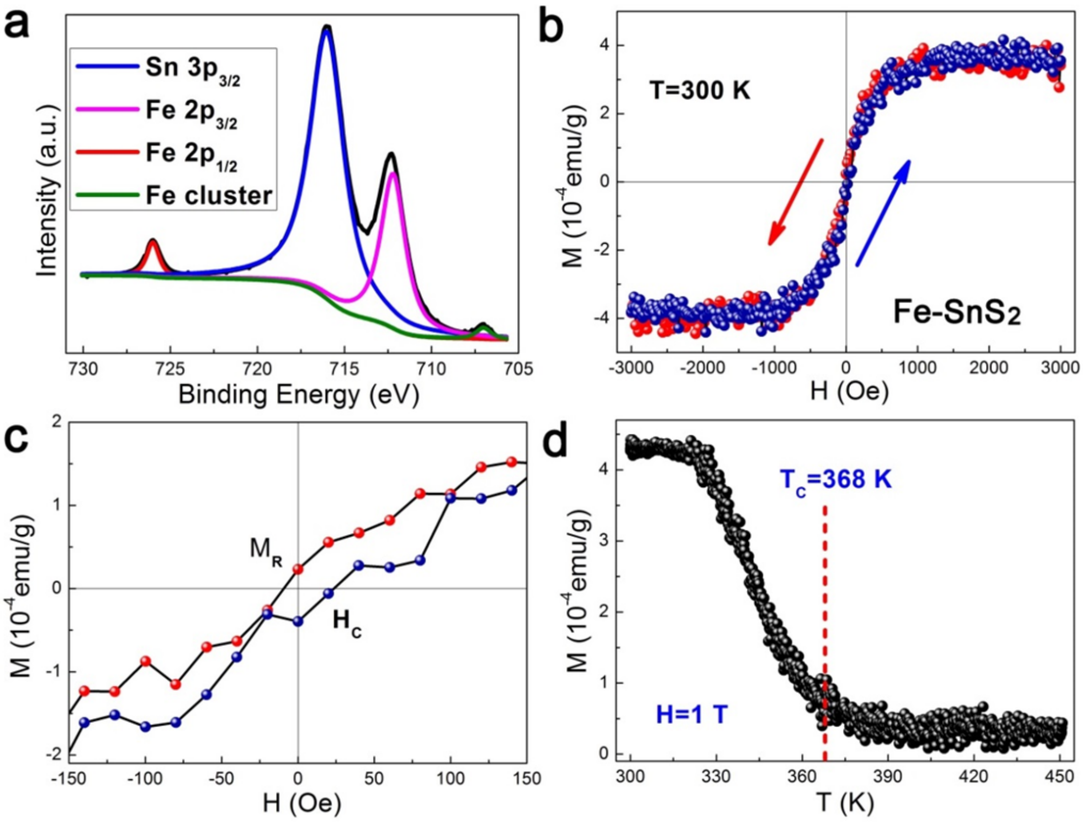

Supplementary Figure 11. (a) XPS spectra of Fe-SnS_2 with Fe cluster. (b) Magnetic hysteresis loops Fe-SnS_2 with Fe cluster at 300 K using VSM. (c) The expanded view of the loop in (b). (d) Magnetization as a function of temperature for the Fe-SnS_2 with Fe cluster from 300 to 450 K. The applied magnetic field is 1T. The applied magnetic field is perpendicular to the sheet ($H_{\perp}$).

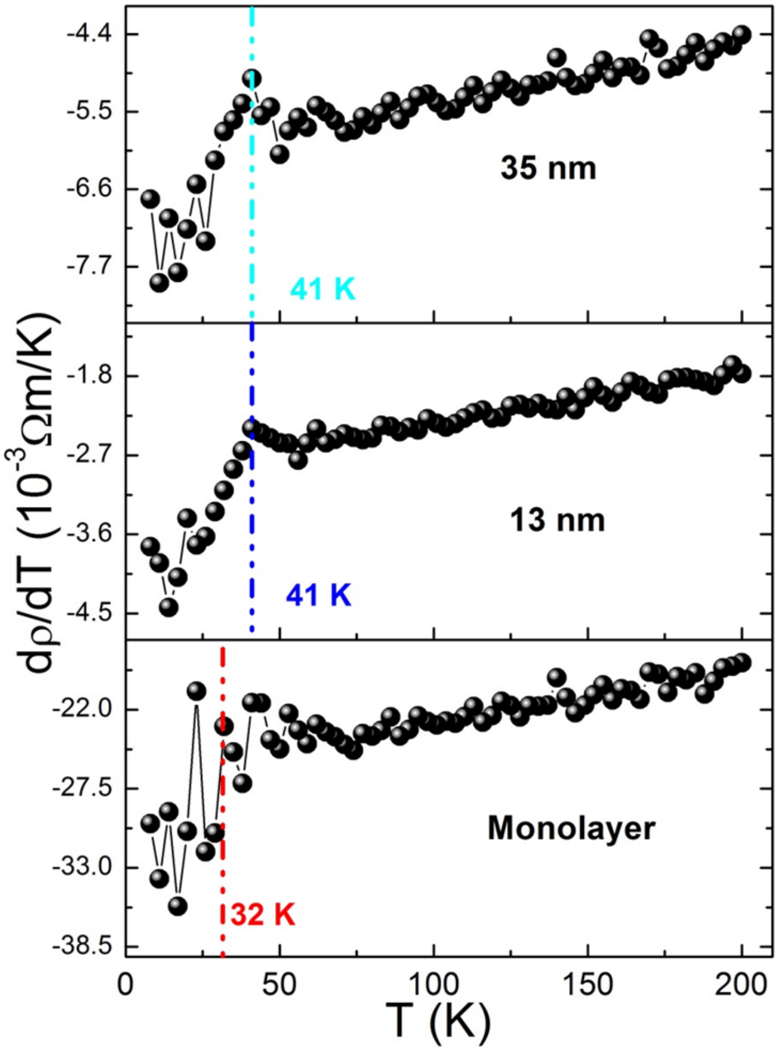

Supplementary Figure 12. Resistivity and temperature derivative of the measured resistivity of $Fe_{0.021}Sn_{0.979}S_2$ nanosheets with the thickness of monolayer, 13 and 35 nm.

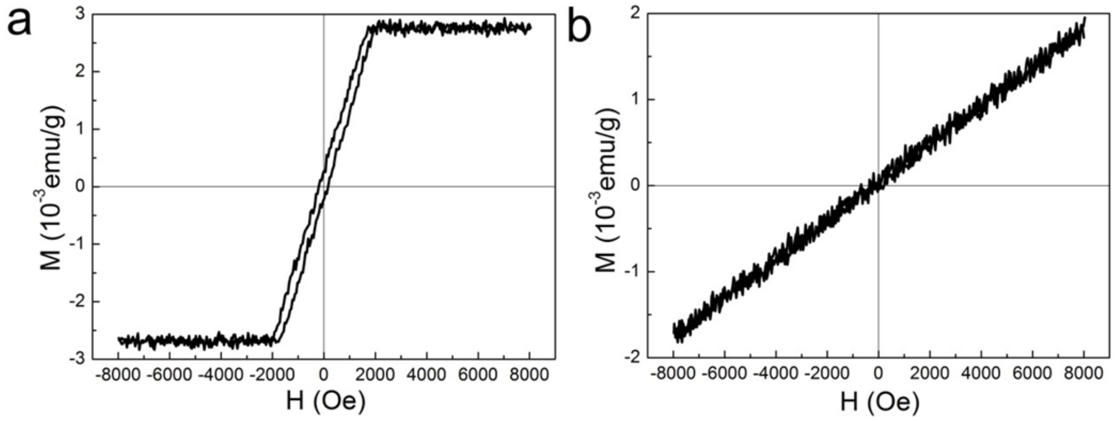

Supplementary Figure 13. (a) and (b) Magnetic hysteresis loops for $Fe_{0.015}Sn_{0.985}S_2$ and $Fe_{0.011}Sn_{0.989}S_2$ at 2 K, respectively. The applied magnetic field is perpendicular to the sheet.

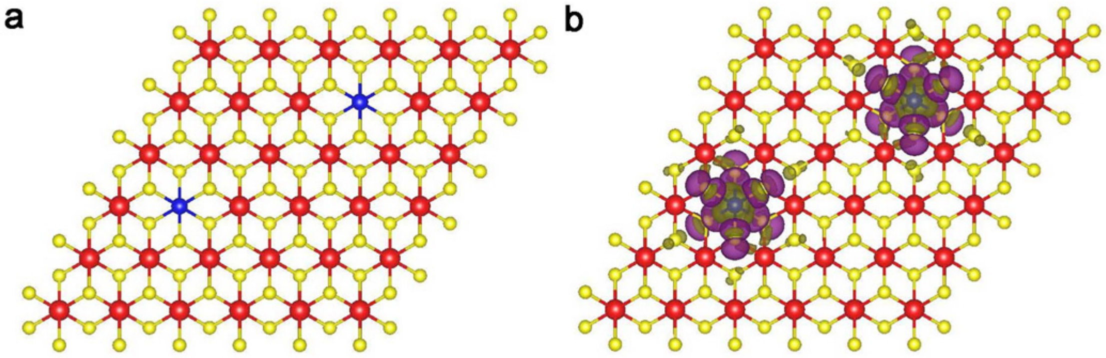

Supplementary Figure 14. (a) 108-atom supercell structure of Fe-SnS_2. (b) The corresponding isosurface plots showing the spin charge density. The isosurface value is taken at 0.001 eÅ^{-3}. The calculation is carried out by DFT+U method.

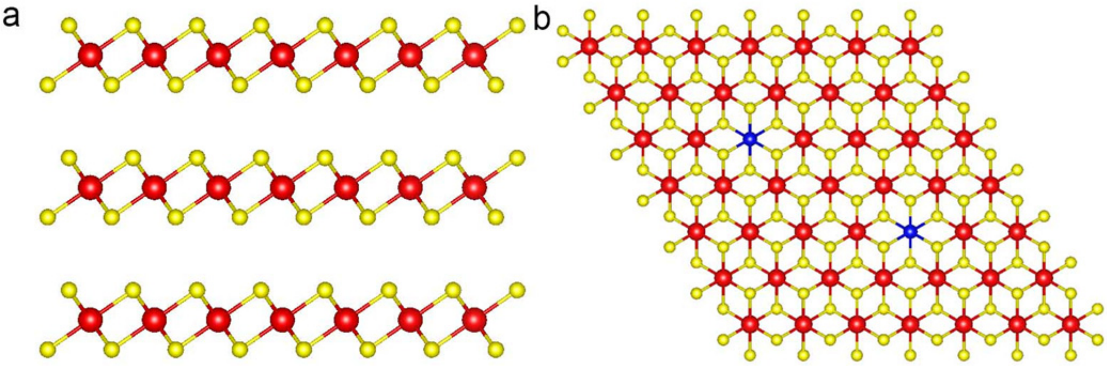

Supplementary Figure 15. Atomic structure of bulk Fe-SnS_2. (a) side view (b) top view of the Fe-SnS_2. The calculation is carried out by DFT+U method.

Supplementary Table 1. Summary of the field-effect performance transistor parameters including electron mobility (μ), conductivity (σ) of the transistors based on our Fe-SnS_2 and SnS_2 monolayers on Al_2O_3/Si substrate, and the previously reported SnS_2 few-layer on SiO_2/Si substrate.

Parameters	$Fe_{0.021}Sn_{0.979}S_2$ monolayer	$Fe_{0.015}Sn_{0.985}S_2$ monolayer	$Fe_{0.011}Sn_{0.989}S_2$ monolayer	SnS_2 monolayer	SnS_2 few-layer[1,2]
μ ($cm^2V^{-1}s^{-1}$)	8.15	6.24	4.76	3.43	5
ON/OFF ratio	7.3×10^6	6.1×10^6	6.3×10^6	1.3×10^6	4.7×10^6
R (mAW^{-1})	206	\	\	83	8.8

Supplementary Table 2. DFT calculated effective mass at conduction band bottom of SnS_2 monolayer and Fe-SnS_2 monolayer

Effective mass	$m^*_{M\text{-}\Gamma}$	$m^*_{M\text{-}K}$
SnS_2 monolayer	$0.82m_0$	$0.54m_0$
Fe-SnS_2 monolayer	$0.74m_0$	$0.47m_0$

Supplementary Table 3. Magnetic parameters of $Fe_{0.021}Sn_{0.979}S_2$ single crystal

Parameters	M_S (10^{-3} emug^{-1})	H_C (Oe)	M_R (10^{-3} emug^{-1})
Perpendicular direction ($H_\perp$)	3.49	400	2.45
Parallel direction ($H_\parallel$)	3.45	130	0.46

Supplementary Note 1. Discussion of optoelectronic property of Fe-SnS_2. The mechanism of photocurrent generation is clarified in **Supplementary Fig. 9**. The absorption of photons generates electron-hole pairs that are separated by the external applied bias, generating a photocurrent and reducing the electrical resistance of the semiconductor. The photocurrent (I_p) can be estimated with the following formula:

$$I_\mathrm{p} = \Gamma \cdot \eta \cdot e \frac{\tau_\mathrm{p} \cdot \mu \cdot V}{L^2} \quad (1)$$

Where Γ is the number of absorbed photons per unit time, η is the efficiency of the conversion of the absorbed photons to electrons, e is the electron charge, τ_p is photogenerated carrier lifetime, μ is the mobility and V is the source-drain bias, L is the length of the transistor channel. The mobility of Fe-SnS_2 is larger than that of SnS_2 for the smaller effective mass, and the photogenerated carrier lifetime of Fe-SnS_2 should be smaller than that of SnS_2 for the impurity levels that promoting the electron-hole recombination. The response time is strongly related with the photogenerated carrier lifetime and the response time of Fe-SnS_2 is larger than that of SnS_2 under the same condition (**Supplementary Fig. 8**).

Supplementary Note 2. Magnetic property of $Fe_{0.027}Sn_{0.973}S_2$. The proportion of the source material is important in the experiment, and we have added the content of Fe source in the growth of the crystal (the stoichiometric proportions of Sn, S, and $FeCl_3$ powders is about 10:30:2), and found Fe cluster in the sample by XPS (**Supplementary Fig. 11a**). XPS result shows that the content of Fe is 2.7 % in the Fe-SnS_2 with Fe cluster. Different from the Fe-SnS_2 without Fe cluster, this sample has an additional Fe *2p* peak at 706.8 eV, exhibiting Fe cluster with 0 valence. Hysteretic behavior of Fe-SnS_2 with Fe cluster is clearly observed at 300 K, consistent with ferromagnetism, having H_C and M_R of 2.3×10^{-5} emug^{-1} and 22.4 Oe, respectively (**Supplementary Figs. 11b** and **c**). The Curie temperature T_C is about 368 K (**Supplementary Fig. 11d**). Thus Fe cluster will increase the Curie temperature to above the room temperature, and the previous Curie temperature of 31 K should come from the pure Fe-SnS_2 rather than the Fe cluster.

Supplementary Reference

1 Huang, Y. et al. Tin disulfide-an emerging layered metal dichalcogenide semiconductor: materials properties and device characteristics. *ACS Nano* **8**, 10743-10755 (2014).

2 Su, G. et al. Chemical vapor deposition of thin crystals of layered semiconductor SnS_2 for fast photodetection application. *Nano Lett.* **15**, 506-513 (2015).
